# The Value of Thin Layer Cytology in Cancer Patients’ Pericardial Effusions

**DOI:** 10.3390/curroncol32060306

**Published:** 2025-05-26

**Authors:** Christos Lafaras, Evdokia Mandala, Kyranna Lafara, Ioannis Kalafatis, Thomas Achladas, Vasiliki Koukoulitsa, Chrysoula Gouta, Theodora Tsiouda, Soultana Skevoudi

**Affiliations:** 1 Cardiology-Oncology Department, Theagenion Cancer Hospital, PC54623 Thessaloniki, Greece; jkal71@gmail.com (I.K.); vkoukoul@yahoo.gr (V.K.); 2 Division of Hematology, Forth Department of Medicine, School of Medicine Aristotle University of Thessaloniki, PC54623 Thessaloniki, Greece; eudokiamandala@gmail.com (E.M.); kyralafa@gmail.com (K.L.); axladtom@gmail.com (T.A.); 3 Department of Cytopathology, Theagenion Cancer Hospital, PC54623 Thessaloniki, Greece; ch.gouta@gmail.com (C.G.); tskevoudi@gmail.com (S.S.); 4 Pulmonology-Oncology Department, Theagenion Cancer Hospital, PC54623 Thessaloniki, Greece; doratsiouda@yahoo.gr

**Keywords:** metastatic pericardial effusions, cardiac tamponade, Thin Layer Cytology, liquid based cytology, ThinPrep, immunocytochemistry

## Abstract

**Objective:** Early diagnosis and treatment of metastatic pericardial disease are crucial to prevent the life-threatening complication of cardiac tamponade. Thin Layer Cytology (TLC), a widely adopted technique in cytology, has gained significant acceptance for most specimens. Our study aimed to assess the utility of TLC in diagnosing metastatic neoplasms and their origins in pericardial effusions, as well as monitoring response to chemotherapy. **Methods:** We examined 184 pericardial fluids collected by pericardiocentesis and processed using the ThinPrep liquid-based technique. Various immunocytochemical markers were used to determine the site of metastatic neoplasms. We also evaluated the response to therapy in 53 patients with lung and breast cancer. **Results:** Out of 184 specimens, 113 pericardial fluids were diagnosed as positive for malignancy, while 71 were negative. Twenty-three cases of unknown primary site were included in the total positive cases. Ninety cases positive for malignancy had a known primary site of origin, including 31 lung carcinomas, 22 breast carcinomas, 10 ovarian carcinomas, 6 T-cell lymphomas, 3 urinary bladder carcinomas, 4 renal carcinomas, 5 adenocarcinomas of the colon, 5 prostate carcinomas, 2 parotid adenocarcinomas, and 2 melanomas. Regarding the 53 cases with chemotherapy treatment, the cytologic examination of pericardial fluid showed a remarkable reduction in neoplastic burden after the third dose of cisplatin or thiotepa instilled into the pericardial cavity. ThinPrep provided excellent preservation of cytomorphological features, high cellularity per slide, and a clear background. This comprehensive analysis provides crucial information about the types and distribution of cancerous cells present in the samples. **Conclusions:** Thin Layer Cytology (TLC) is a valuable diagnostic tool for detecting metastatic pericardial malignancy. It allows the examination of exfoliated cells from the pericardial fluid, providing crucial information for diagnosis, management, and monitoring the acute responsiveness to intrapericardial chemotherapy. Immunocytochemistry (IHC) can identify specific markers for various types of cancer, enabling a more accurate diagnosis and guiding further treatment decisions.

## 1. Introduction

Pericardial disease in cancer patients can arise from various pathologic factors, including idiopathic conditions, primary or metastatic neoplasms, radiotherapy, conventional chemotherapeutic agents, targeted therapy, and immunotherapy. Metastatic pericardial effusion is a well-recognized complication in patients with advanced malignancies and can progress slowly or rapidly to potentially fatal cardiac tamponade. The incidence of metastatic malignancy in pericardial effusions ranges from 2% to 31% [1,2]. The most common primary sites associated with pericardial effusion are the lung in men (56%) and the breast in women (14%). Early detection and diagnosis of pericardial disease in cancer patients are essential. Imaging modalities like echocardiography, CT scans, and MRI can aid in identifying pericardial effusions and assessing their extent.

Treatment of malignant pericardial effusions should be individualized, considering the patient’s condition, tumor type, success rates, risks of various modalities, and local availability and expertise. The most frequent treatment modalities involve draining the pericardial fluid either by percutaneous pericardiocentesis under echocardiographic or fluoroscopic guidance or by a surgical subxiphoid approach. This allows for fluid sample collection, pericardial drainage, and pericardial biopsy. Additional percutaneous procedures include percutaneous balloon pericardiotomy (single or double balloon), which creates a direct pleuropericardial communication, allowing fluid drainage into the pleural space. Pericardioscopy permits visualization of the pericardial sac, enabling the physician to take targeted biopsy specimens from the epicardial and pericardial layers, avoiding epicardial vessels and increasing the probability of obtaining disease-specific results in experienced centers [3,4,5,6].

A percutaneous pericardioperitoneal shunt offers a minimally invasive alternative to surgical shunting under local anesthesia and imaging guidance (fluoroscopy or ultrasound) to place a catheter between the pericardium and the peritoneum without requiring open surgery. The procedure is suitable for patients who are poor candidates for surgery due to comorbidities or those who prefer less invasive options. It can also be used in cases where rapid symptom relief is necessary, but surgery poses too high a risk.

The creation of a pericardial window via left mini-thoracotomy and/or video-assisted thoracoscopy is a safe and effective approach in the surgical treatment of malignant cardiac tamponade, allowing the malignant pericardial effusion to drain from the intrapericardial space into the chest cavity. A surgical pericardio-peritoneal shunt, under general anesthesia, allows excess fluid to drain from the pericardial into the abdominal cavity, where it can be absorbed more readily. It is typically indicated for patients with recurrent malignant effusions that do not respond well to less invasive treatments and require careful postoperative monitoring for complications such as infection or catheter blockage. Finally, pericardiectomy, a major surgical procedure, although very effective, appears disadvantageous due to its relatively high mortality rate (13%) and is indicated only in cases of constrictive pericardial disease. Both approaches are viable alternatives depending on patient-specific factors such as overall health status, anatomy, risk profiles, recovery, and personal preferences [1,2,4,5].

Cytological examination of pericardial fluid plays a crucial role as it not only confirms the malignant etiology but also helps to identify the primary tumor site in cases of metastatic disease [2,3,4,5,7,8].

ThinPrep, also known as Thin Layer Cytology, is a method used for preparing cytology slides that allows for improved visualization and detection of abnormal cells. In the context of pericardial effusions, ThinPrep has been shown to enhance the sensitivity of detecting metastatic neoplasms compared to conventional cytology techniques.

In cases where conventional cytodiagnosis may not provide a definitive answer regarding the presence of malignancy in pericardial effusions, immunocytochemistry can be employed as an adjunctive tool. By using specific antibodies to detect markers associated with different types of cancer, immunocytochemistry can help differentiate between benign and malignant cells with greater accuracy [1,2]. The study highlights the significance of accurate cytodiagnosis in pericardial effusions, especially in patients with a history of cancer or those with unknown primary tumors. By identifying the origin of metastatic neoplasms through ThinPrep and immunocytochemistry, healthcare providers can tailor treatment strategies accordingly [7,9,10,11,12,13]. Furthermore, the intrapericardial infusion of chemotherapeutic agents based on the chemosensitivity profile of the primary tumor holds promise in preventing the recurrence of pericardial effusion. This personalized approach not only improves patient outcomes but also enhances survival rates and overall prognosis [10,14,15,16].

## 2. Materials and Methods

After Institutional Review Board approval, the electronic pathology database at Theagenion Cancer Hospital was queried for PE specimens collected from January 2003 to January 2024. Patient demographics, cytological diagnosis, past medical history, comorbid conditions, and the volume of pericardial specimens were recorded for each case. Our study included 184 pericardial fluids.

All patients experienced clinical and/or subclinical cardiac tamponade, defined as the decompensated phase of cardiac compression resulting from increased intrapericardial pressure, with clinical symptoms and signs including dyspnea, elevated jugular venous pressure, hypotension, tachycardia, and pulsus paradoxus. In the initial stages of hemodynamic impairment due to pericardial effusion, echocardiographic findings indicated heart chamber compression, including early diastolic collapse of the right ventricle, late diastolic collapse of the right atrium, abnormal ventricular septal motion, exaggerated respiratory variability (>25%) in mitral inflow velocity, inspiratory decrease and expiratory increase in pulmonary vein diastolic forward flow, respiratory variation in ventricular chamber size, aortic outflow velocity, and inferior vena cava plethora. After the diagnosis of cardiac tamponade was confirmed, the patients were admitted to the intensive care unit, where they underwent subxiphoid pericardiocentesis under electrocardiographic, echocardiographic, and hemodynamic guidance. Penetration into the pericardial sac was performed with the patient positioned at a 45-degree angle, using a soft, multihole, J-shaped 7F catheter (Seldinger technique). For the pericardiocentesis and drainage, we used the PeriVac system (Boston Scientific Technologies, Marlborough, MA, USA). An immediate increase in stroke volume, a reduction in intrapericardial and atrial pressures, and a separation between right and left filling pressures resulted in the restoration of adequate cardiac output in all patients. The catheter remained in the pericardial cavity for 6 to 10 days.

Among the patients who underwent subxiphoid pericardiocentesis, 4 out of 184 (2.17%) encountered supraventricular tachycardia, 8 out of 184 (4.34%) atrial fibrillation (5 self-limiting, 2 confronted by amiodarone, 1 requiring DC shock), 5 out of 184 (2.71%) non-sustained ventricular tachycardia, 2 out of 184 (1.08%) transient sinus bradycardia (vasovagal event), and 1 out of 184 patients (0.5%) experienced superficial right atrial puncture (no need for surgical intervention). There were 10 out of 184 patients (5.43%) with recurrent cardiac tamponade requiring secondary intervention; 6 underwent a second pericardiocentesis and 4 a surgical pleuro-pericardial window via left minithoracotomy.

It should be emphasized that all the specimens were collected by pericardiocentesis, and 30 mL of the cell population lying on the bottom of the pericardiocentesis sac was centrifuged. The pallet was then inserted into a ThinPrep bottle, prepared using ThinPrep technique (Liquid Based—Cytyc Corp., Boxborough, MA, USA) and stained by Papanicolaou method. ThinPrep involves a new monolayer automated technique for the collection and preparation of cytologic specimens. This method requires direct transfer of cells into a preservative solution. The cell suspension is gently dispersed, homogenizing the cell population. The cells are then automatically collected on a polycarbonate filter and transferred to a glass slide in a well-circumscribed small area.

Cytological examination employs immunocytological methods as an important tool for diagnosis (positive or negative), differential diagnosis, and primary site location. In positive specimens, immunocytological techniques are often essential for identifying the type, locating an unknown primary tumor, or verifying a known one based on imaging and the patient’s medical history. The most important step in differential diagnosis requires identifying the specific entity and type, a procedure that depends partly on the cytological features of the neoplastic cells but mainly on thorough immunocytochemical examination. In pericardial fluid, as well as in peritoneal and pleural fluid, distinguishing between reactive mesothelial cells and neoplastic cells is sometimes very challenging, a problem often overcome through immunocytochemistry. For this reason, negative and ambiguous specimens are quite commonly subjected to immunocytochemical examination, a method that guides the cytologist toward the most accurate diagnosis.

**Figure 1:**
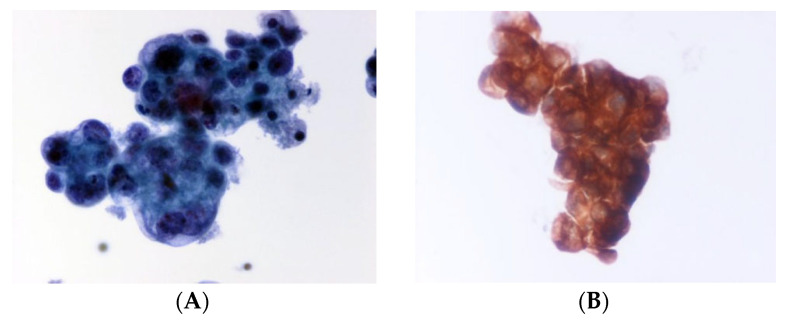
Cytomorphological image indicative of metastatic adenocarcinoma (ACA) of lung in pericardial effusion. High cellularity with numerous atypical cells, neoplastic cells as clusters or singly dispersed, nuclear pleomorphism. (**A**). Papstain (reveals nuclear details—membrane irregularities, chromatin pattern, and intranuclear invaginations, ThinPrep ×400. (**B**). NapsinA (Immunohistochemistry for Nap-A, a functional aspartic proteinase, as a marker for primary lung ACA. ThinPrep ×400.

**Figure 2:**
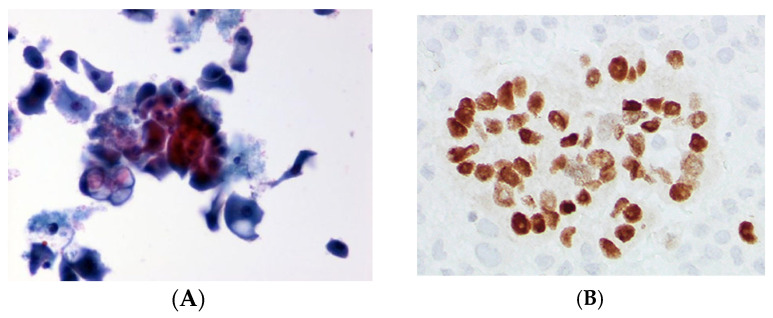
Cytomorphological image of metastatic ductal adenocarcinoma (invasive breast carcinoma, NST) of the breast in pericardial effusion. (**A**). Papstain, ThinPrep ×400. (**B**). GATA3 (nuclear staining) positive, positive in ductal adenocarcinoma, ThinPrep ×400.

**Figure 3:**
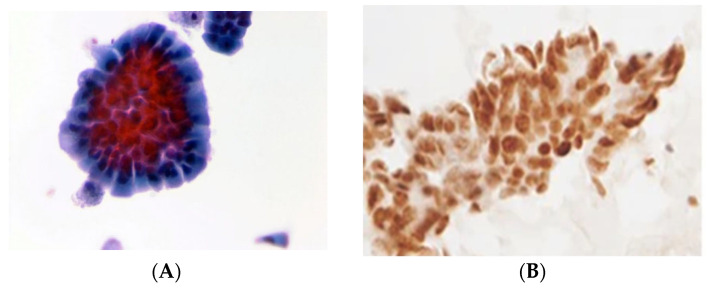
Cytomorphological image of metastatic adenocarcinoma of the colon in pericardial effusion. (**A**). Papstain, ThinPrep ×400. (**B**). CDX2 positive in adenocarcinoma of colon, ThinPrep ×400.

In each case, a panel of selective immunocytochemical markers was used to verify the origin of the metastatic tumor (Table 1, Figure 1, Figure 2 and Figure 3). The differential diagnosis of malignant tumors of the lung, which, together with breast neoplasms, are the most common causes of malignant pericardial effusion, includes squamous cell carcinoma, adenocarcinoma, and small cell carcinoma. Therefore, immunocytochemical markers such as TTF1, p40/p63, napsin A, and neuroendocrine markers (synaptophysin, chromogranin, CD56) play a crucial role in both locating the primary origin but also in identifying the type of lung carcinoma among these three entities. Likewise, in breast cancer, the differential diagnosis lies between invasive breast carcinoma, NST (formerly known as ductal breast carcinoma), and invasive lobular breast carcinoma and is facilitated by the use of e-cadherin immunocytochemical testing. Both types are positive for GATA3, CA15-3, and GCDFP-15, while examination for markers such as ER, PR, and c-erbB2 is considered imperative for the following decision-making of the treatment. In ovaries, on the other hand, low- and high-grade carcinoma is the most commonly found tumor in pericardial fluid cytological testing, with markers such as PAX8, CA125, WT1, ER, and PR validating the diagnosis, while the presence or absence of p53 mutation gives an insight of the grade and possible prognosis. Other primary tumors (non-Hodgkin’s T cell lymphomas, urothelial carcinoma, renal cell carcinoma, adenocarcinoma of the colon and prostate) can be easily detected with highly specific and/or sensitive immunocytochemical markers such as CD3, GATA3, CD10, CDX2, and PSA. CD20 staining, although not positive in any of our cases, combined with CD3, is fundamental for the differential diagnosis between B and T cell lymphomas. Last but not least, cytopathologists have at hand a full panel of markers for the detection of metastatic melanoma (HMB-45, Melan A, Sox10, S-100) and its differential diagnosis from other malignancies. Pankeratin (AE1/AE3) is positive in all epithelial tumors and is used mainly to rule out non-epithelial neoplasms (lymphomas, melanomas). In case of epithelial origin and especially in highly undifferentiated/dedifferentiated tumors, in which the cytological characteristics are not indicative of a certain type and possible primary location, the CK7 and CK20 expression profile, in combination with other markers, is very helpful since various organs show a distinct CK7/CK20 pattern (Table 2). The specific immunocytochemical staining for each of the aforementioned markers can be seen in Table 3.

After the completion of the procedures (ThinPrep monolayer automated technique, Papanicolaou staining, immunocytochemistry), the specimen is stored in a ThinPrep bottle at room temperature for at least three months and can be retrieved for further examination, if needed, up to a year after collection. ThinPrep slides (both immunocytochemical and Papanicolaou stains) are kept in the slide archive of the Cytopathological Laboratory of our hospital for 20 years.

Besides immunocytochemistry, the cytomorphological features of neoplastic pericardial effusions frequently guide diagnosis. Adenocarcinomas, including lung, colon, and prostate adenocarcinoma, as well as ductal breast carcinoma NST, share some common cytological characteristics. Neoplastic cells are usually organized in small glandular or spheroid clusters (the latter being very characteristic of invasive breast carcinoma NST) or, less commonly, in acinar and papillary formations. Cell overlapping may be observed. Neoplastic cells display malignant characteristics, including nuclear membrane irregularity, enlarged and pleomorphic nuclei, nuclear hyperchromasia, dense chromatin, increased N/C ratio, and prominent nucleoli. The cytoplasm may often be vacuolated or foamy. The background usually contains necrotic debris and inflammatory infiltrates, including lymphocytes, neutrophils, and macrophages. Lobular breast carcinoma, on the other hand, consists predominantly of discrete cells and cord-like arrangements (“Indian file” arrangement). Another significant neoplastic category is squamous cell carcinoma, with the lung being the most common primary location in our study. Neoplastic cells are usually polygonal with abundant eosinophilic cytoplasm and hyperchromatic atypical nuclei. In contrast, small cell carcinoma of the lung consists of small cells with hyperchromatic nuclei and scant cytoplasm, exhibiting characteristic cell molding. These features pose a challenge in differential diagnosis with B and T cell lymphomas. Lastly, melanoma cells are recognized by prominent macronucleoli, intranuclear cytoplasmic inclusions, binucleated cells, intracytoplasmic brown melanin pigment, and, in some cases, spindle cell morphology [1,2,14].

After the malignant etiology of the pericardial fluid was confirmed by cytological examination, local chemotherapy, based on the chemosensitivity of the primary tumor, was administered directly into the pericardial space. We use strict criteria in the diagnosis of malignancy, including positive cytology, elevated neoplastic markers in the pericardial fluid, and/or pericardial masses/implantations depicted by echocardiography (Table 4). Treatments were chosen on an individual basis by the multidisciplinary team (oncologists, pulmonologists, cardiologists) according to clinical presentation (neoplastic burden, previous therapies, performance status, and comorbidities), evolving knowledge and experience, and recent guidelines. Each patient gave informed consent to the treatment. A total of 9/40 (22.5%) patients with lung cancer and 7/29 (24.1%) with breast cancer received only systemic chemotherapy. It should be emphasized that local chemotherapy was delivered to the emptied pericardial sac. After relief of cardiac tamponade, systemic chemotherapy was periodically administered to patients with no contraindications. Clinical and echocardiographic evaluations were performed after intrapericardial chemotherapy and repeated monthly thereafter. Treatment was considered successful when no recurrence of pericardial effusion was observed and pericardial implantations either reduced or disappeared in repeated echocardiographic evaluations.

In 53 patients with a known primary tumor (lung or breast), more than one sample was evaluated (one sample before and three after local chemotherapy). After cytological examination and confirmation of the presence of neoplastic cells in the pericardial fluid, cisplatin (10 mg in 20 mL normal saline) for lung cancer patients and Thiotepa (30 mg in 20 mL of sterile water) for breast cancer patients were instilled into the pericardial cavity for three consecutive days [5]. After the first, second, and third doses of intrapericardial chemotherapeutic agent administration, cytological examination was performed, and the neoplastic burden was evaluated [12,13]. Clinical and echocardiographic follow-up examinations were conducted monthly.

Survival was obtained through active follow-up based on the verification of each patient’s vital status. All analyses involved were performed using SPSS software (SPSS Inc., Chicago, IL, USA), version 28, and *p* values below 0.05 were considered statistically significant.

## 3. Results

Out of 184 specimens, 113 pericardial fluids were diagnosed as positive for malignancy, while 71 were negative. Twenty-three cases of unknown primary site were included in the total positive cases. Ninety cases positive for malignancy had a known primary site of origin, including 31 lung carcinomas, 22 breast carcinomas, 10 ovarian carcinomas, 6 T-cell lymphomas, 3 urinary bladder carcinomas, 4 renal carcinomas, 5 adenocarcinomas of the colon, 5 prostate carcinomas, 2 parotid adenocarcinomas, and 2 melanomas.

In all cases, ThinPrep technology produced slides with a true monolayer of cells spread on the same plane, preserving cellular integrity and maintaining a clean background. Moreover, this technique provided high cellular density and facilitated the easy detection of malignant cells. Using a panel of selective immunocytochemical markers, the 23 cases of unknown primary site were finally diagnosed as metastatic carcinomas from the ovary (5), breast (7), lung (9), and melanoma (2).

**Figure 4:**
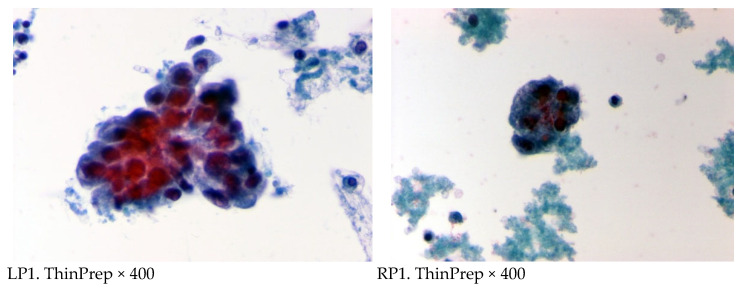

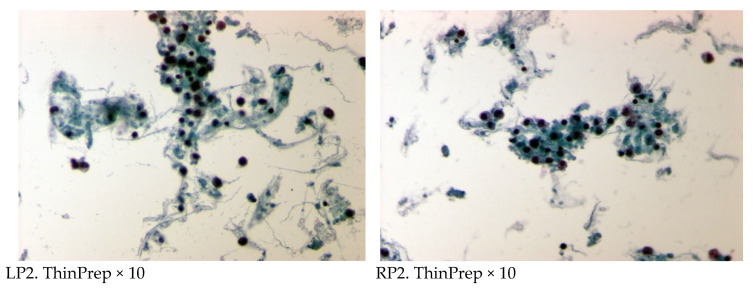
Cytological examination of pericardial fluid before (**left panel**, LP1, LP2) and after the 3rd dose of intrapericardial administration of cisplatin (**right panel**, RP1, RP2) in lung cancer patients with malignant cardiac tamponade. A reduced neoplastic burden is depicted by decreased and smaller sample-sized groups of malignant cells in these smears.

**Figure 5:**
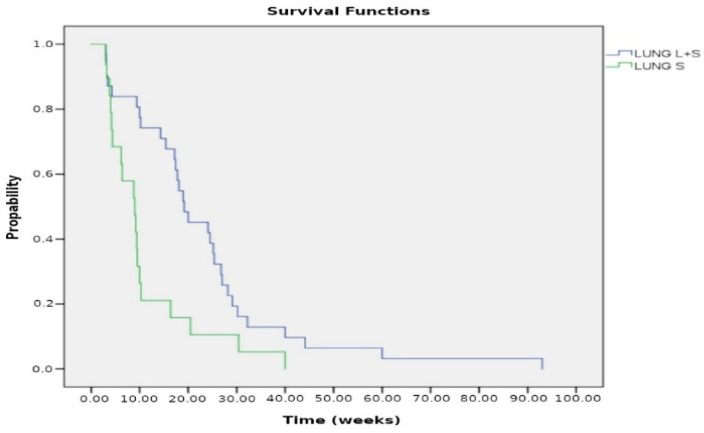
Kaplan–Meier curves for patients with lung cancer comparing combined local and systemic chemotherapy (L + S) versus systemic chemotherapy alone (S). The median survival was 19.2 weeks for the L + S treatment compared to 9 weeks for the S treatment; *p* < 0.005.

**Figure 6:**
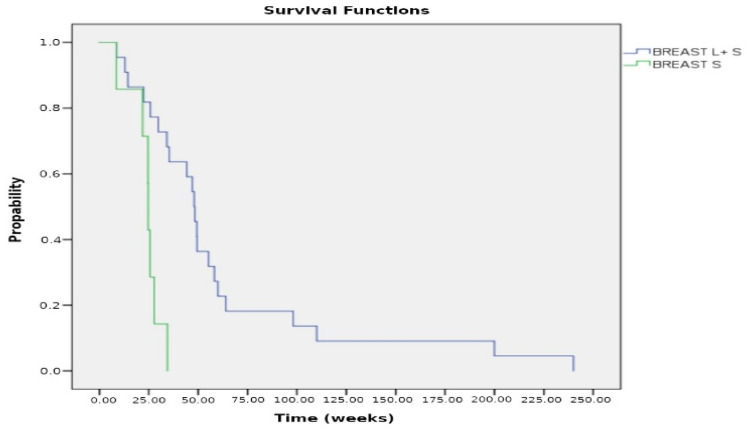
Kaplan–Meier curves for patients with breast cancer who received combined chemotherapy: Local plus systemic (L + S) vs. systemic chemotherapy (S) alone. The median survival was 48 weeks for the L + S treatment compared to 24.7 weeks for the S treatment; *p* = 0.001.

**Figure 7:**
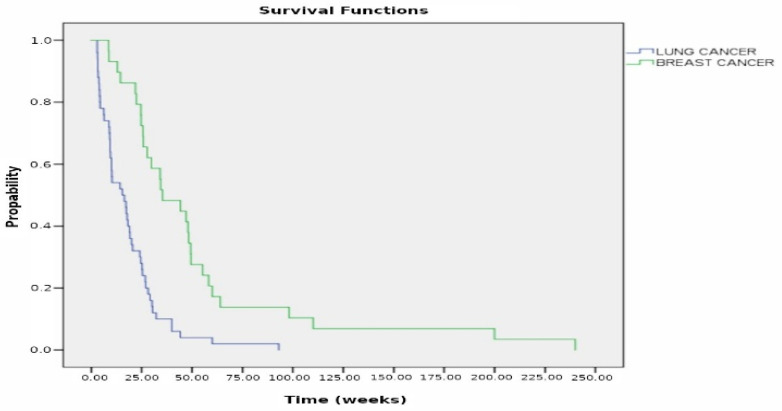
Kaplan–Meier curves of observation in patients with breast cancer vs. lung cancer. The median survival was 15.4 weeks for lung cancer vs. 35.4 weeks for breast cancer; *p* < 0.00005.

Regarding the 53 cases with intrapericardial chemotherapy, the cytological examination of pericardial fluid showed a remarkable reduction in neoplastic burden after the third dose of cisplatin or thiotepa, instilled into the pericardial cavity (Figure 4). The median survival of lung cancer patients was 4.8 months (19.2 weeks) (range 3–93 weeks) for those who underwent systemic plus intrapericardial chemotherapy versus 2.3 months (19.2 weeks) (range 3–40 weeks, *p* < 0.005) for those without local treatment. The median survival of patients with breast cancer after systemic plus intrapericardial thiotepa instillation was 12 months (48 weeks), whereas survival without local chemotherapy vs. 6.17 months (24.7 weeks) with the systemic treatment; *p* = 0.001 (Figure 5 and Figure 6). The median survival was 15.4 weeks for lung cancer vs. 35.4 weeks for breast cancer; *p* < 0.00005 (Figure 7). Deaths were attributed to progression of the oncologic disease, generalized carcinomatosis, and/or cachectic metastatic disease in all patients. No patient died of recurrent cardiac tamponade.

## 4. Discussion

Although cardiac tamponade (CT) is often the result of malignant cells in the pericardium, the etiology of symptomatic pericardial disease is not always due to malignancy. Certain chemotherapeutic agents, such as anthracyclines, cyclophosphamide, and taxanes, have been implicated in causing pericardial disease due to their direct effect on the pericardium, potentially leading to cardiac tamponade. While targeted therapies are generally considered less toxic than traditional chemotherapy due to their specificity for cancer-related pathways, they are not devoid of cardiovascular side effects and can contribute indirectly to pericardial effusions. Immunotherapies, such as checkpoint inhibitors, carry risks related to immune-mediated adverse events affecting the myocardium and/or pericardium, potentially leading to pericardial effusion. Radiation therapy directed at thoracic malignancies can inadvertently affect cardiac structures, resulting in pericardial effusion, related to the radiation dose and concomitant antineoplastic therapy [1,2,8]. Neoplasms with early metastases to mediastinal lymph nodes may present as CT, which explains the predominance of lung cancer in this setting. Cardiac metastases may occur by lymphatic or hematogenous dissemination, as well as by direct extension from the mediastinum [16]. The early diagnosis and treatment of metastatic pericardial disease are of great importance due to the danger of fatal cardiac tamponade. Moreover, the local application of chemotherapeutic agents can significantly improve cancer patients’ survival. The acute treatment of malignant cardiac tamponade involves prompt and complete removal of pericardial fluid by pericardiocentesis or surgical intervention (pericardial fenestration). Recurrence of malignant pericardial effusion and subsequent tamponade is extremely frequent (40–70%) [17,18]. Survival of patients with malignant tumors and large pericardial effusions mainly depends on the patient’s condition, presence of metastases, response to additional systemic treatment, local treatment, and prevention of cardiac tamponade recurrence [17].

Furthermore, the early detection and precise definition of the origin of malignant cells in pericardial effusions play an important role in the choice of appropriate therapeutic procedures, especially in patients with two primary neoplasms. According to bibliographic data, cytology and pericardial biopsy have sensitivities of 90% and 56%, respectively [8]. The lower sensitivity of pericardial biopsy is likely due to the fact that tumors generally spread from the mediastinal and subepicardial lymphatics to the visceral pericardium, pericardial fluid, and finally to the parietal pericardium [2].

The cytological examination of pericardial fluid is valuable in identifying the origin of metastatic pericardial tumors. The hemorrhagic background and the low or moderate cellularity of pericardial smears can cause difficulties in precise diagnosis. Recently, these problems have been mitigated by the use of Thin Layer Cytology, which provides better cytomorphology and a cleaner background, allowing for easier interpretation compared to conventional methods. Moreover, Thin Layer Cytology facilitates the application of immunocytochemistry due to the possibility of creating archival material. Additionally, immunocytochemistry consumes fewer reagents due to the lower background and smaller area of the ThinPrep slide containing the target cells [19].

In this study, the smears from pericardial effusions were usually of moderate cellularity with mesothelial or reactive cells and a variable number of malignant cells. A total of 71 out of 184 specimens (38.58%) were negative for malignancy, significantly lower than the average 50% reported in the literature. This discrepancy is possibly due to the hospital being a cancer-oriented referral center in Northern Greece. Additionally, this could be attributed to radiation, chemotherapeutic agents, immunotherapy, inflammation, or idiopathic causes. Moreover, the presence of a multihole, J-shaped catheter in the pericardial cavity causes aseptic inflammation, increasing the production of inflammatory cells. Reducing the neoplastic burden may affect recognizing and targeting malignant cells present in the effusion. Immediate cytodiagnosis is of great value and should include the larger portion of the pericardial effusion drained as soon as possible. Knowledge of the positivity or negativity of pericardial fluid is of great significance for patient management. Our data confirm that the most commonly encountered tumor types are lung and breast. The cytological diagnosis of malignancy was easy in most cases when clinical information was provided in association with ThinPrep and immunocytochemical application.

The diagnostic dilemmas encountered in some cases focus on differentiating atypical reactive cells from mesotheliomas or metastatic adenocarcinomas and detecting the tumor type, especially in patients with unknown primary tumors.

Immunocytochemistry plays a key role in accurate cytodiagnosis in these cases. A review of the literature shows that the origin of some metastatic neoplasms with an unknown primary often involves frequent sites such as the lung, breast, melanoma, and non-Hodgkin’s lymphoma [8,16,18,20,21], as well as rare sites such as the gastrointestinal tract [21,22], urinary bladder [1,18,23,24], and parotid gland [25,26]. Reactive mesothelial cells and malignant mesothelioma cells can sometimes be challenging to differentiate due to their similar appearance under the microscope. Reactive mesothelial cells and malignant mesothelioma cells can sometimes be challenging to differentiate due to their similar appearance under the microscope. To distinguish between the two, a panel of immunocytochemical markers is often used, with BAP1 being the most important. Its retained nuclear stain indicates reactive mesothelial proliferations, while mesothelioma cells are characterized by their loss of expression. Additionally, p53 and EMA are positive in malignant mesothelioma cells but negative in reactive mesothelial cells. EMA, in particular, is useful in distinguishing mesothelioma, which displays strong membranous staining, from adenocarcinoma of any origin, where cytoplasmic staining is typically observed [21].

Regarding the evaluation of the response to therapy in our study, the longest survival was associated with breast cancer. This indicates that breast cancer is more responsive to chemotherapy compared to lung carcinoma. This observation is supported by extensive research documented in authoritative sources, which delve into the biological, clinical, and therapeutic aspects of these cancers. First, breast cancer and lung carcinoma are fundamentally different at the cellular and molecular levels. Second, breast cancer has benefited from advancements in personalized medicine, where treatments are tailored based on specific genetic markers such as HER2 status, which can predict responsiveness to certain targeted therapies in addition to conventional chemotherapeutic agents. On the contrary, lung carcinoma, while also subject to personalized approaches with drugs targeting EGFR mutations or ALK rearrangements and immunotherapy, generally shows lower overall response rates to chemotherapy compared to breast cancer. Finally, the survival outcomes for breast cancer patients have improved dramatically, whereas lung carcinoma typically has a much poorer prognosis for all stages combined. A review of the literature shows that our data aligns with previous studies. [1,8,12,13,24].

It is well known that intrapericardial chemotherapy according to chemosensitivity of the primary tumor represents a valuable approach for managing malignant pericardial effusions in addition to systemic conventional treatment [26,27,28,29,30]. Hilar lymphatic drainage plays a role in the early manifestation of malignant pericardial effusion during cancer. After intrapericardial chemotherapy, high concentrations of the substance exist in lymphatic vessels, reducing the local neoplastic burden, improving drainage, and consequently preventing recurrence of pericardial effusion. This localized approach, in addition to achieving higher concentrations of the drug delivered directly to the pericardial cavity, minimizes systemic exposure and potential side effects compared to intravenous administration, allowing for effective treatment while reducing toxicity. Alternatively, intrapericardial sclerotherapy is used to manage recurrent malignant pericardial effusions (PE). The administration of sclerosing agents, such as tetracycline derivatives like doxycycline, bleomycin, mitoxantrone, and talc, induces fibrosis and obliteration of the pericardial cavity to prevent re-accumulation of PE. However, their use should be tailored based on individual patient characteristics and clinical scenarios after a thorough evaluation by a multidisciplinary team [31,32,33]. In our experience, therapeutic intervention for malignant pericardial effusion using intrapericardial chemotherapy is preferable to local sclerotherapy, as it is a curative rather than palliative approach, even in frail oncologic patients. We achieve not only the prevention of recurrence of pericardial effusion but also address pericardial implantations, leading to either an apparently normal pericardium or a thickened pericardium without constriction.

Reviewing the literature, this is one of the first studies to confirm the effectiveness in neoplastic burden of pericardial effusion after intrapericardial instillation of cisplatin and/or thiotepa in patients suffering from lung and breast cancer, respectively (Figure 4). By using TLC, clinicians can assess the effectiveness of intrapericardial chemotherapy in real-time by evaluating changes in cell populations before and after treatment.

Fortunately, the International System for Reporting Serous Fluid Cytology (TIS) was developed to address the variability in diagnostic practices for serous effusions, which can arise from both neoplastic and non-neoplastic conditions. The primary goal of TIS is to provide a uniform framework for reporting serous fluid cytology results, thereby improving diagnostic accuracy and interobserver agreement. Its adoption globally underscores its validity as a classification scheme that enhances diagnostic accuracy, facilitates better clinical decision-making, and ultimately improves patient care [34,35].

The present study has some limitations. First of all, the study is retrospective and observational, based on treatments chosen and tailored for each patient by individual oncologists, with potential bias. Secondly, the duration is long and does not include all modern therapeutic strategies (targeted therapy, immunotherapy), particularly in certain types of cancer that affect the metastatic process and overall survival. Consequently, there is heterogeneous management due to the rarity of malignant cardiac tamponade.

## 5. Conclusions

Thin Layer Cytology has been shown to enhance the diagnostic accuracy of metastatic pericardial fluids, surpassing the effectiveness of conventional methods. This technique offers a significant advantage in terms of reproducibility, making it easier to conduct immunocytochemistry on the same sample. The cytologic examination not only aids in detecting malignancy but also provides crucial insights into the response to therapy for patients with malignant pericardial effusion.

Furthermore, both Thin Layer Cytology and immunocytochemistry play a pivotal role in refining the identification of the origin of metastatic pericardial tumors. Early detection of malignancy and timely intervention are vital contributors to the long-term survival of cancer patients. These methods are invaluable tools for guiding management and treatment planning for these patients.

In summary, this study contributes valuable insights into the effectiveness of intrapericardial chemotherapy for treating neoplastic burdens associated with malignant pericardial effusions. The combination of this targeted therapy with Thin Layer Cytology offers a promising approach for improving patient outcomes in cases complicated by cancer-related fluid accumulation.

## Figures and Tables

**Table 1:** Causes of cardiac tamponade/cytological examination of pericardial effusion (immunocytochemical markers).

Malignant (+)	Number of Patients	Immunocytochemical Markers	Malignant (−)	Number of Patients
Primary Tumors			Etiology	
Lung Squamous cell carcinoma Adenocarcinoma Small cell carcinoma	40	p40, p6,CK5/6 3TTf1, NapsinA TTF1, synaptophysin chromogranin CD56, INSM1	Chemotherapy induced	12
Breast Invasive breast carcinoma, NST Invasive lobular breast carcinoma	29	Ca15-3, GATA3, GCDFP-15(BRST2), e-cadherin, ER, PR, c-erbB2 GATA3, CA15-3, GCDFP-15 (BRST2), ER PR, c-erbB2	Immunotherapy induced	2
Ovarian Serous carcinoma Mucinous carcinoma	15	PAX8, CA125, WT1, ER, PR, p53 PAX8, CK-20	Radiation induced	4
T-cell lymphomas	6	LCA, CD3, CD20, CD15, CD45	Uremia	3
Urinary Bladder	3	CATA3, P63, P40, CK-20	Inflammatory	14
Renal	4	PAX8, CAIX, CD10, EMA	Idiopathic	34
Colon	5	CDX2, CK20, CA19-9	Chylopericardium	2
Prostate	5	PSA, AMACR		
Parotid	2	EMA, CATA3, c-erbB2, 34βE12		
Melanomas	4	HMB-45, S-100, Sox10 Melan-A		

**Table 2:** CK20/CK7 patterns of expression in various primary locations in our study.

CK20+, CK7+	CK20−, CK7−	CK20+, CK7−	CK20−, CK7−
Urinary bladder Ovary (mucinous)	Prostate	Colon	Breast Lung Ovary (serous) Parotid Kidney

**Table 3:** Immunocytochemical markers used in our study classified according to the type of staining.

Markers	Type of Staining
INSM1, CDX2, PAX8, WT1, p53, ER, PR SOX10, GATA3, p40, p63	Nuclear
Keratins, 34βE12, synaptophysin, chromogranin, napsin A, HMB45, CA 19-9 Melan A, AMACR, GCDFP-15, PSA, EMA	Cytoplasmic
CD56, LCA, CD3, CD20, e-cadherin c-erbB2, CD10, CAIX, CA125	Membranous
S-100	Nuclear and Cytoplasmic
CA 15-3	Membranous and Cytoplasmic

**Table 4:** Co-existing pericardial implantations (metastases) in patients with malignant cardiac tamponade.

Malignant Cardiac Tamponade		Total Pericardial Metastases	Synchronous Pericardial Metastases	Metachronous Pericardial Metastases
Primary Tumor	Patients	Patients (%)	Patients (%)	Patients (%)
Lung cancer	40	18 (45%)	14 (35%)	1 (10%)
Breast cancer	29	9 (31%)	7 (24.10%)	2 (6.80%)
Ovarian	15	3 (20%)	2 (13.30%)	1 (6.66%)
T-cell lymphomas	6	2 (33.30%)	2 (33.30%)	0
Urinary Blooder	3	1 (33.30%)	0	0
Renal cancer	4	1 (25%)	1 (25%)	0
Colon	5	2 (40%)	1 (20%)	1 (20%)
Parotid	2	1 (50%)	1 (50%)	0
Melanomas	4	2 (50%)	2 (50%)	0

## Data Availability

The original contributions presented in this study are included in the article. Further inquiries can be directed to the corresponding author.

## Abbreviations

TTf1: Transcription Termination Factor 1, NAP-1:NapsinA, Ca15-3: Carbohydrate antigen15-3, GATA3: GATA-binding protein 3, GCDFP-15(BRST2): Gross cystic disease fluid protein-15, LCA: leukocyte common antigen, CD: cluster designation or cluster of differentiation CD3, CD10, CD20:, CD15:, CD45:, Ca-125: Cancer antigen-125, PAX8: paired box gene8, Wt1: Wilms’ tumor1, CK-20: Cytokeratin-20, P63:protein63, P40: protein40, CAIX: Carbonic Anhydrase IX, Vimentin: type III intermediate filament (IF) protein, PSA: prostatic specific antigen, AMACR: Alpha-methylacyl-CoA racemase, CK7: Cytokeratin7, Ca19-9: Carbohydrate antigen19-9, CDX2: Caudal-related homeobox transcription factor 2, CEA: Carcinoembryonic antigen, EMA: Epithelial membrane antigen, HMB-45: Human melanoma black antibody-45, S-100: protein saturated-100.
